# Feasibility and acceptability of monitoring personal air pollution exposure with sensors for asthma self-management

**DOI:** 10.1186/s40733-021-00079-9

**Published:** 2021-09-05

**Authors:** Sherrie Xie, Jessica R. Meeker, Luzmercy Perez, Whitney Eriksen, Anna Localio, Hami Park, Alicia Jen, Madison Goldstein, Akua F. Temeng, Sarai M. Morales, Colin Christie, Rebecca E. Greenblatt, Frances K. Barg, Andrea J. Apter, Blanca E. Himes

**Affiliations:** 1 Department, of Biostatistics, Epidemiology and Informatics, University of Pennsylvania, 402 Blockley Hall, 423 Guardian Drive, Philadelphia, PA 19104 USA; 2 Pulmonary, Allergy and Critical Care Division, Perelman School of Medicine, University of Pennsylvania, Philadelphia, PA 19104 USA; 3 Mixed Methods Lab, Perelman School of Medicine, University of Pennsylvania, Philadelphia, PA USA; 4 Spelman College, Atlanta, GA USA; 5 Florida State University, Tallahassee, FL USA

**Keywords:** Air pollution, Personal monitors, Personal exposure, Asthma, Particulate matter

## Abstract

**Background:**

Exposure to fine particulate matter (PM_2.5_) increases the risk of asthma exacerbations, and thus, monitoring personal exposure to PM_2.5_ may aid in disease self-management. Low-cost, portable air pollution sensors offer a convenient way to measure personal pollution exposure directly and may improve personalized monitoring compared with traditional methods that rely on stationary monitoring stations. We aimed to understand whether adults with asthma would be willing to use personal sensors to monitor their exposure to air pollution and to assess the feasibility of using sensors to measure real-time PM_2.5_ exposure.

**Methods:**

We conducted semi-structured interviews with 15 adults with asthma to understand their willingness to use a personal pollution sensor and their privacy preferences with regard to sensor data. Student research assistants used HabitatMap AirBeam devices to take PM_2.5_ measurements at 1-s intervals while walking in Philadelphia neighborhoods in May–August 2018. AirBeam PM_2.5_ measurements were compared to concurrent measurements taken by three nearby regulatory monitors.

**Results:**

All interview participants stated that they would use a personal air pollution sensor, though the consensus was that devices should be small (watch- or palm-sized) and light. Patients were generally unconcerned about privacy or sharing their GPS location, with only two stating they would not share their GPS location under any circumstances. PM_2.5_ measurements were taken using AirBeam sensors on 34 walks that extended through five Philadelphia neighborhoods. The range of sensor PM_2.5_ measurements was 0.6–97.6 μg/mL (mean 6.8 μg/mL), compared to 0–22.6 μg/mL (mean 9.0 μg/mL) measured by nearby regulatory monitors. Compared to stationary measurements, which were only available as 1-h integrated averages at discrete monitoring sites, sensor measurements permitted characterization of fine-scale fluctuations in PM_2.5_ levels over time and space.

**Conclusions:**

Patients were generally interested in using sensors to monitor their personal exposure to PM_2.5_ and willing to share personal sensor data with health care providers and researchers. Compared to traditional methods of personal exposure assessment, sensors captured personalized air quality information at higher spatiotemporal resolution. Improvements to currently available sensors, including more reliable Bluetooth connectivity, increased portability, and longer battery life would facilitate their use in a general patient population.

**Supplementary Information:**

The online version contains supplementary material available at 10.1186/s40733-021-00079-9.

## Introduction

Air pollution is harmful to health and contributes substantially to the global disease burden [1, 2]. While air pollution is detrimental to all people regardless of health status, its effects disproportionately impact those with underlying conditions such as asthma. Moderate increases in fine particulate matter (PM_2.5_), nitrogen dioxide (NO_2_), ozone, carbon monoxide and traffic-related air pollution (TRAP) are known to trigger exacerbations in adults and children with asthma [3–6], and recent studies have linked early- and mid-life exposure to PM_2.5_, NO_2_, and TRAP to greater asthma prevalence [7–11]. Yet despite the role that air pollution plays in asthma onset and morbidity, accurately quantifying personal pollution exposure for individuals remains a challenge.

Air pollution exposure estimates for research studies are often based on measurements taken by stationary regulatory monitors, such as those operated by the U.S. Environmental Protection Agency (EPA). While these monitors are highly accurate and well-suited for ensuring compliance to federal air quality standards, their utility for capturing individual-level pollution exposure is limited for a few key reasons: 1) Due to their relative sparsity, monitor locations rarely coincide with the locations that exposure takes place (e.g., home, work or school), and thus, an individual’s exposure to air pollution can only be measured *indirectly* through spatial interpolation techniques, such as inverse distance weighted interpolation and kriging, or statistical methods, such as land-use regression modeling; 2) regulatory monitors offer limited temporal resolution (e.g., hourly averages in the case of particulate matter monitors), which may lead them to miss transient spikes in pollution levels; 3) indirect methods of exposure assessment typically estimate exposure for a single location per individual, such as their location of residence, place of work [12] or school [13], which does not capture exposures that occur while people are at different locations or during regular activities like commuting and errands; 4) methods that rely on outdoor regulatory monitors can only capture *ambient* pollution concentrations rather than exposures occurring inside the home or other indoor settings, which is a significant limitation given that most individuals in industrialized nations spend > 90% of their time indoors [14].

Low-cost portable pollution sensors are accessible environmental monitoring devices that can be carried or worn by individuals during their usual activities. Because they measure pollution levels directly and in real-time, they could enable health providers and researchers to monitor individual-level exposures and empower patients to manage their personal exposure to pollutants beyond what is possible with regulatory monitors [15, 16]. While their accuracy is not as high as that of regulatory or research-grade monitors, researchers have demonstrated the practicality of using lower cost pollution sensors to assess indoor air quality [17–21] and pollution levels within commute microenvironments [22–24]. Early personal exposure studies using sensors have also highlighted the disproportionate impact of commuting and cooking to total pollution exposure experienced by individuals [25–32]. However, few studies have used personal sensors to monitor pollution exposure in patients with respiratory diseases [26, 33]. Because they can be used to aid in self-management of symptoms, personal pollution sensors may be especially helpful for individuals with asthma, and particularly for those living in disadvantaged neighborhoods, which tend to have higher air pollution levels [34]. Philadelphia is a city largely comprised of Environmental Justice areas as defined by the state of Pennsylvania (i.e., census tracts where 20 percent or more of individuals live in poverty and/or 30 percent or more of the population is minority), and the region has been consistently ranked among the most challenging places to live with asthma by the Asthma and Allergy Foundation of America [35, 36]. In this study, we aimed to: 1) understand the acceptability and preferences for using personal pollution sensors among adults with persistent asthma who reside in the Greater Philadelphia Area, 2) assess the feasibility of using sensors to measure PM_2.5_ exposure, and 3) compare sensor PM_2.5_ measurements to standard estimates obtained using measurements taken by regulatory monitors.

## Methods

### Study population

Individuals were eligible for the study if they were Penn Medicine asthma patients aged 18 years or older with a prescription for inhaled corticosteroids and residence in the Greater Philadelphia Area (Philadelphia, Delaware, Chester, Montgomery, Bucks, Camden Gloucester or Burlington counties). Patients were contacted by phone, screened to determine if they met eligibility criteria, and invited to enroll. Fifteen patients were recruited and asked about their demographic characteristics and asthma history. To determine each patient’s level of asthma control, interviewers administered the asthma control questionnaire (ACQ) during a phone interview. The ACQ is a validated measure of asthma control derived from questions regarding patient-reported symptoms during the day and night, limitations on daily activities, rescue bronchodilator use, and lung function (i.e., forced expiratory volume in 1 s [FEV_1_]) [37–39]. Due to the infeasibility of assessing lung function over the phone, interviewers administered a shortened version of the ACQ, which included all items except FEV_1_, although previous studies have found good agreement between full and shortened versions of the ACQ [38, 39]. All versions of the ACQ yield a score ranging between 0 (totally controlled) and 6 (severely uncontrolled), and level of asthma control was defined using previously validated cut-points: [40] patients with an ACQ score ≤ 0.75 were classified as having *well-controlled asthma*, patients with an ACQ score between 0.75–1.5 (not inclusive) were classified as having *partially controlled asthma*, and patients with an ACQ score ≥ 1.5 were classified as having *inadequately controlled asthma*.

### Semi-structured interviews

Experienced interviewers from the University of Pennsylvania Mixed Methods Laboratory conducted all interviews using a standardized script developed for this study (Supplementary Materials). Semi-structured interviews were conducted over the phone and recorded to identify preferences and acceptability of use of personal pollution sensors. Participants were asked about their attitudes and preferences towards using a personal pollution sensor, as well as their privacy preferences regarding the dissemination of personal sensor data. Recordings of interviews were transcribed, de-identified, and thematically analyzed in accordance with a grounded theory framework using the qualitative software program NVivo 11. A codebook representing the key ideas that emerged from the interviews was developed after eight interviews had been conducted and was iteratively refined in the coding of an additional five interviews. Content saturation appeared to have been reached after coding interviews 1–12: participant responses were well clarified by existing codes, and no new themes were emerging. To ensure saturation, an additional three interviews were conducted, in which no new themes were identified. Two reviewers established strong interrater reliability, κ = 0.98, with four (26%) of the interviews. Of the remaining eleven interviews, eight were recorded by one reviewer and three were recorded by the other. Because English was the preferred language of all participants, interviews were conducted in English only.

### Low-cost sensor field trials

The AirBeam, developed by the non-profit environmental health organization HabitatMap, is a low-cost portable air monitoring device that measures PM_2.5_, temperature and humidity (Supplementary Figure 1). Its performance has been validated by the EPA, the South Coast Air Quality Management District and other research groups who have demonstrated moderate-to-good agreement (*R*^2^ = 0.43–0.71) between uncalibrated AirBeam PM_2.5_ measurements and EPA reference methods across a diverse range of outdoor environmental conditions [41–45]. In addition, the AirBeam offers a visual interface that provides users with real-time feedback on air quality via the Android AirCasting app. In the summer (Jun-Aug) of 2018, student research assistants used AirBeam devices to take PM_2.5_ measurements while walking in the West Philadelphia and Center City neighborhoods of Philadelphia. Walking routes were selected to cover geographical areas with diverse characteristics, but also with consideration for route accessibility with start/end point at the University of Pennsylvania and safety of research personnel. Walks were taken primarily in the cooler morning and evening hours and avoided on excessively hot, humid or rainy days. After AirBeam sensors were synced to an Android mobile device via Bluetooth, sensor measurements were recorded using the HabitatMap AirCasting app and subsequently exported to a local data server. Before they were deployed outdoors, sensors were tested indoors to assess basic functionality and evaluate the correlation of PM_2.5_ measurements recorded by different sensors while a controlled source (i.e., stick of burning incense) emitted particles for 1–3 h; measurements were highly correlated (Pearsons’ *r* > 0.98) for all between-sensor comparisons (Supplementary Figure 2). No formal sensor calibration was performed. Sensor measurements were recorded at 1-s intervals and included the following variables: PM_2.5_, temperature, and relative humidity, along with timestamp and GPS coordinates (latitude and longitude) obtained from the paired Android smartphones.

#### Regulatory monitor PM_2.5_ measures

Sensor PM_2.5_ measurements were compared to contemporaneous PM_2.5_ measurements recorded at three nearby regulatory monitoring stations that comprise a part of the EPA’s ambient air monitoring network; measurements taken by these regulatory monitors are henceforth referred to as *EPA measurements*. EPA measurements were obtained from the EPA Air Quality System (AQS) database as raw JSON files via the AQS API and subsequently imported into *R* for analysis [46, 47].

#### Sensor and PM_2.5_ measure data analysis

In contrast to sensor measurements, which were taken once per second, EPA measurements were reported on an hourly basis. Sensor measurements were thus time-matched according to the hour of measurement. For example, all sensor PM_2.5_ measurement taken between 9:00:00 and 9:59:59 EDT were matched to EPA measurements recorded for 9:00 EDT on the same day such that a single measurement reported by an EPA monitor may correspond to many sensor measurements. For each sensor PM_2.5_ measurement, an “EPA estimate” was derived for the GPS location associated with the sensor measurement using time-matched EPA measurements via inverse-distance-squared-weighted interpolation. Maps of individual sensor deployments were created by depicting sampling routes as points shaded according to timestamp, temperature, relative humidity, and PM_2.5_, as well as EPA-interpolated PM_2.5_ estimates for the same time and location. Points were 10-s binned averages mapped at 50% opacity to optimize visibility of spatially proximate data points. A summary map of sensor PM_2.5_ measurements averaged at the block level for data collected across all deployments was created by “snapping” all GPS coordinates to the nearest road segment (block), thereby assigning each data point to the block where sampling was presumed to have taken place. Mean PM_2.5_ for all sensor measurements recorded for each block was subsequently computed and block-level averages were visualized on a map. The same process was repeated for EPA estimates. Block-level sensor means were compared to the block-level means computed using EPA estimates by calculating the difference between these two means for each block to determine whether there were regions where local PM_2.5_ levels were above or below the globally interpolated levels. Analyses were conducted in *R* [47].

The protocol was approved by the University of Pennsylvania Institutional Review Board. All participants provided informed consent over the phone.

## Results

**Fig. 1:**
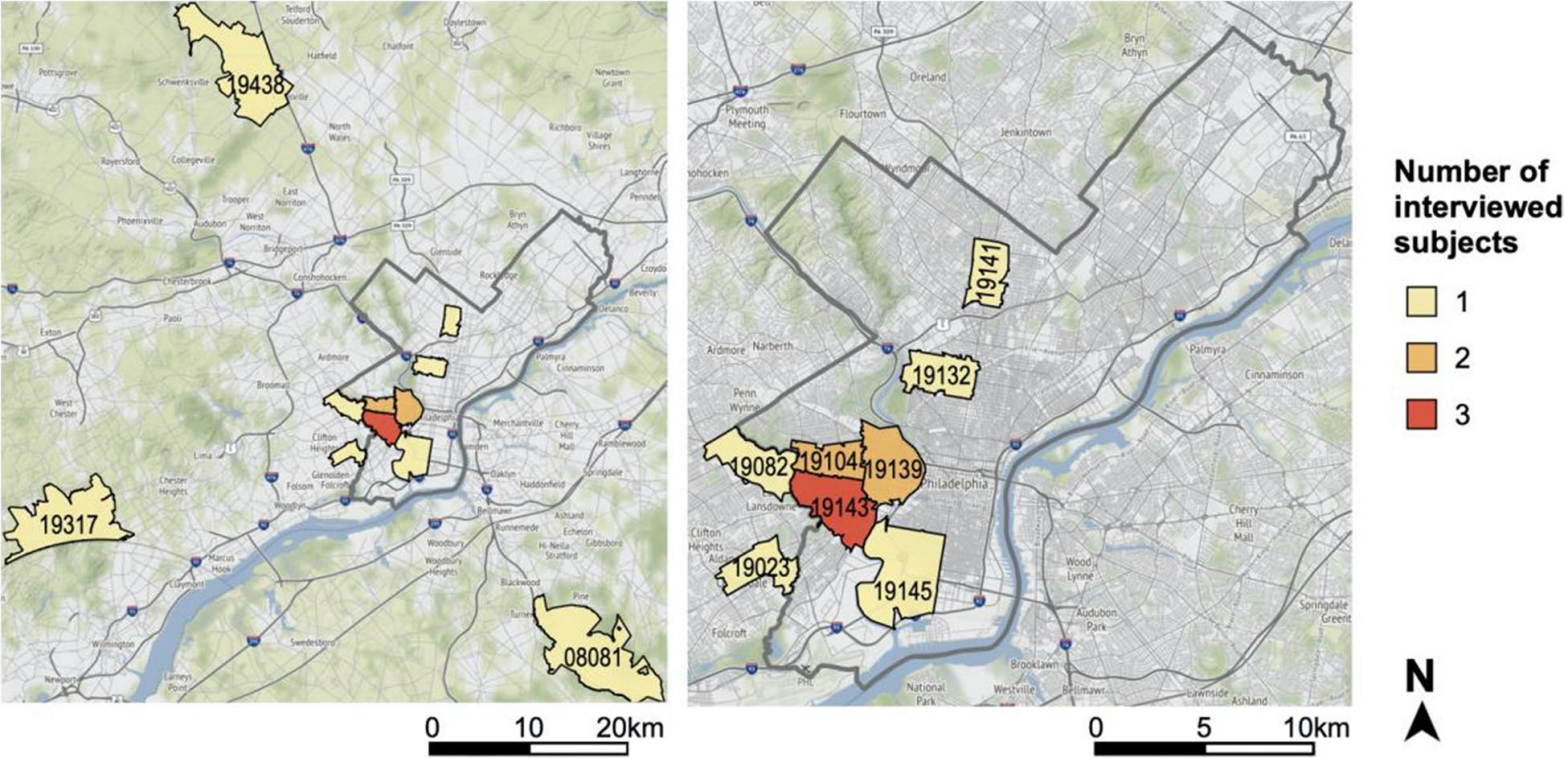
Residential zip code of study participants mapped over the entire Greater Philadelphia Area (left panel) and zoomed in over Philadelphia county (right), with Philadelphia county outlined in grey

The characteristics of the 15 adults with asthma who were interviewed about their preferences and attitudes regarding the use of personal pollution sensors are summarized in Table 1. Reflecting the demographics of patients with persistent asthma who receive care at Penn Medicine, participants were predominantly female and non-Hispanic Black [48] Most patients were middle aged and had either commercial or a combination of commercial and Medicare health insurance. Ten patients resided in Philadelphia County, while five resided in surrounding counties within the Greater Philadelphia Area (Fig. 1). Sixty percent of patients (*n* = 9) had asthma that was inadequately controlled, while forty percent (*n* = 6) had asthma that was either well-controlled or partially controlled.

**Table 1:** Characteristics of study participants (*N* = 15)

Characteristic	n (%)
**Sex**	
Female	13 (87)
Male	2 (13)
**Age in years**	
18–34	3 (20)
35–54	9 (60)
55–74	3 (20)
*Mean (range)*	*46 (23–74)*
**Race/ethnicity**	
Non-Hispanic Black	11 (73)
Non-Hispanic White	2 (13)
Hispanic White	1 (7)
Declined to answer	1 (7)
**Health insurance**	
Medicaid	8 (53)
Commercial ± Medicare	5 (33)
Medicare only	2 (13)
**Asthma control status (ACQ score)**	
Well-controlled (≤ 0.75)	3 (20)
Partially controlled (> 0.75 and < 1.5)	3 (20)
Inadequately controlled (≥ 1.5)	9 (60)

All participants stated they would use a personal air pollution sensor, though the consensus was that the device should be small (watch- or palm-sized), inconspicuous and light. Some of the participants wanted the device to resemble jewelry, while others preferred a small standalone device for in-home use only. One-third of participants wanted the device to augment an existing technology that they habitually used and had on them (e.g., a mobile phone or smartwatch) so that they would not have to carry around an additional device. In terms of device features, participants wanted to receive real-time actionable information regarding the air quality they were being exposed to, and to be able to program the device to help them avoid their personal asthma triggers.

With regard to privacy preferences, participants were generally unconcerned about sharing sensor data, including their GPS coordinates. Specifically, eight participants expressed a lack of concern about sharing their location data, recognizing that they would need to share their location history to determine where pollution exposure took place. Five patients expressed hesitation in sharing their location data but stated they would consider doing so under certain conditions, such as if they could limit sharing of their data to researchers and healthcare providers or if they could turn location sharing on and off at their discretion. Two patients stated they would not share their GPS coordinates under any circumstances.

Research staff used AirBeam sensors to take outdoor measurements of PM_2.5_ concentration on 34 walks of 1-to-3-h duration, collecting 117,510 total sensor PM_2.5_ measurements, which were subsequently time-matched to 135 unique measurements of PM_2.5_ concentration measured by nearby regulatory monitors (41–52 measurements per site; Table 2). Sensor PM_2.5_ measurements ranged 0.6–97.6 μg/m^3^ (mean 6.8 μg/m^3^), compared to 0–22.6 μg/m^3^ (mean 9.0 μg/m^3^) measured by nearby monitors. Outdoor sensor measurements showed moderate correlation with estimates derived from EPA measurements (Pearson’s *r* = 0.509; Supplementary Figure 3).

**Table 2:** Comparison of outdoor PM_2.5_ measurements taken by AirBeam sensors vs. time-matched measurements taken by nearby EPA monitors

	**AirBeam**	**EPA-55**	**EPA-57**	**EPA-76**
Federal equivalent method	No	Yes	Yes	Yes
Measurement method	Optical sensor	Beta attenuation monitor	Beta attenuation monitor	Beta attenuation monitor
Measurement interval	1 s	1 h	1 h	1 h
Number of measurements	117,510	52	41	42
PM_2.5_, μg/m^3^, mean (range)	6.8 (0.6, 97.6)	10.3 (2.9, 22.6)	7.1 (0, 11.9)	9.2 (0.1, 19.7)
Data missingness, %	0	1.8	22.6	20.8

**Fig. 2:**
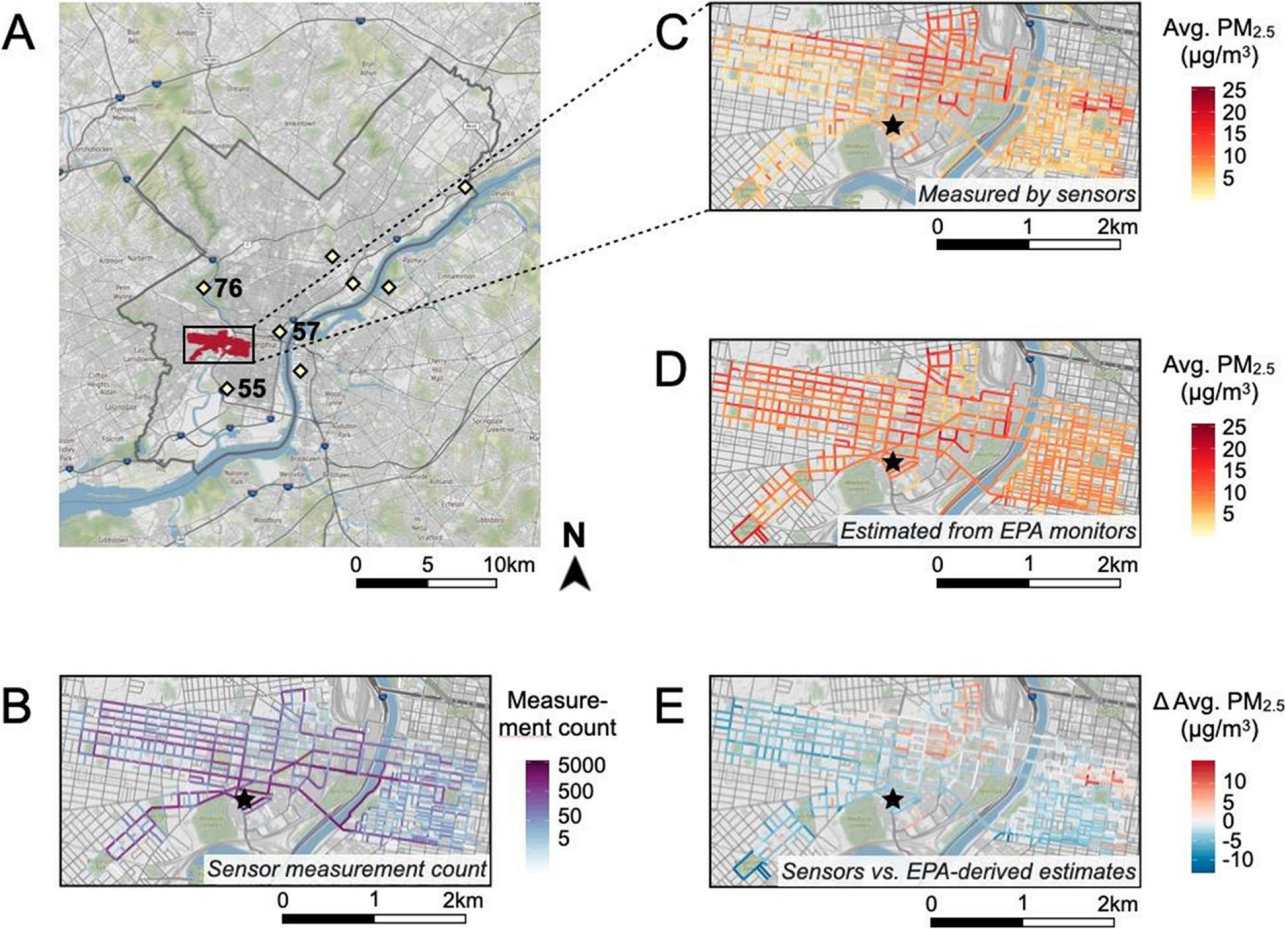
Summary of sensor field trials and comparison with EPA estimates. **A** Regions sampled by AirBeam sensors are indicated in red and circumscribed by the black rectangle, and Philadelphia county is outlined in grey. The locations of EPA monitoring stations are denoted by pale yellow diamonds, with the three closest stations labeled by their site ID’s. Surveyed streets shaded according to **B** the number of sensor PM_2.5_ measurements taken on each block, **C** block-level mean PM_2.5_ measured by sensors, **D** block-level mean PM_2.5_ estimated from time-matched EPA measurements, and **E** difference between PM_2.5_ measured by sensors vs. estimated using EPA measurements. The black star indicates the location of the research lab in panels **B-E**

A visual summary of all sensor deployments and the three most proximate EPA monitoring sites is presented in Fig. 2. Sensors were deployed in a rectangular area encompassing portions of the West Philadelphia (i.e., University City, Spruce Hill, Cedar Park and Kingsessing) and downtown Center City (located east of the Schuylkill River) neighborhoods of Philadelphia (Fig. 2A). The most frequently sampled streets were those located closest to the research lab, followed by those located in neighborhoods to the north (University City) and west (Spruce Hill, Cedar Park and Kingsessing; Fig. 2B). Block-level average PM_2.5_ concentration measured by sensors varied considerably across the study area: areas of low PM_2.5_ concentrations extended through the southwestern regions of West Philadelphia and south Center City and areas of high concentration were located in north University City and north Center City (Fig. 2C). In contrast, block-level average PM_2.5_ concentration estimated using time-matched EPA measurements showed considerably less spatial variation (Fig. 2D). Sensor averages differed from EPA averages by -13 to + 15 μg/m^3^ and mapping the difference between these averages highlighted small areas in Center City and University City with higher-than-expected PM_2.5_ concentrations (Fig. 2E).

**Fig. 3:**
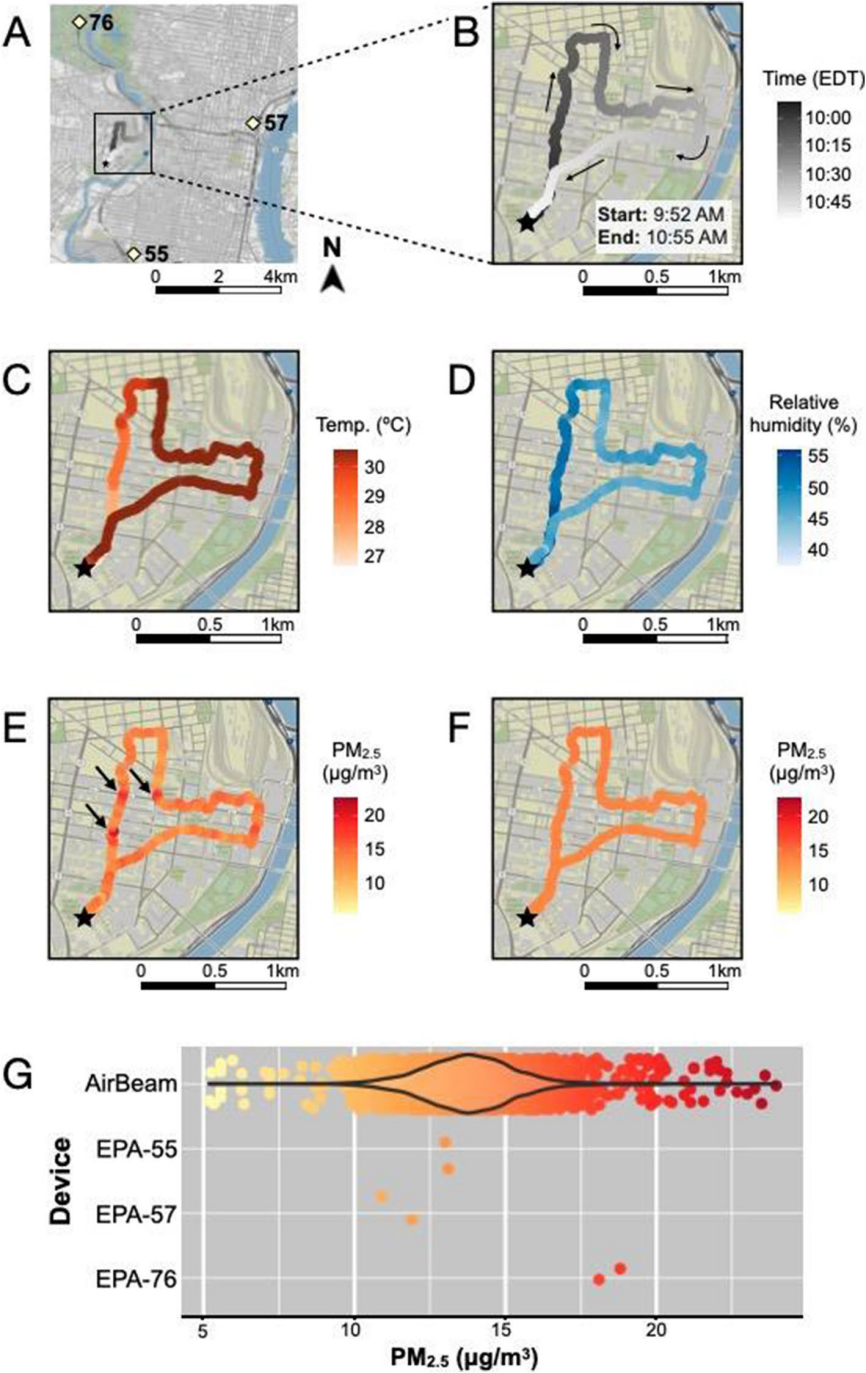
Sample walking route taken by a student research assistant carrying an AirBeam sensor on July 12, 2018. **A** The sampling route is circumscribed by the black rectangle, and the locations of nearby EPA monitoring stations are labeled according to site ID. Close-ups of the route colored according to **B** timestamps, **C** temperature (°C), **D** relative humidity (%), and **E** PM_2.5_ (μg/m^3^) recorded by the sensor, as well as **F** PM_2.5_ (μg/m^3^) estimates derived from EPA measurements via inverse-distance-squared-weighted interpolation. **G** Comparison of sensor and EPA measurements recorded during the sampling frame. The black star indicates the location of the research lab in panels **A-F**

Figure 3 shows an example sampling route taken by research staff carrying an AirBeam sensor on the morning of July 12, 2018. GPS coordinates and timestamps collected during the deployment show that departure from lab was at 9:52 AM, then the researcher headed north through the university campus, east toward the Schuylkill River, before looping back and returning to their starting location at 10:55 AM (Fig. 2A-B). During this walk, the sensor took continuous measurements of temperature, relative humidity, and PM_2.5_ concentration, capturing distinct trends for each variable (Fig. 2C-E). In contrast to temperature, which incrementally increased from 27 °C to a plateau of 30–31 °C and relative humidity, which incrementally decreased from 52% to a plateau of 45–46%, PM_2.5_ concentrations fluctuated during this period (Supplementary Figure 4). Notably, while most PM_2.5_ measurements collected during this deployment ranged between 10–17 μg/m^3^, concentrations spiked (exceeding 20 μg/m^3^) at three timepoints corresponding to when research staff crossed busy street intersections (Fig. 2E; Supplementary Figure 4). In contrast to PM_2.5_ concentrations that were measured directly by the sensor, PM_2.5_ concentrations that were interpolated for the same GPS locations from time-matched EPA measurements showed virtually no variation over time and space (Fig. 2F). This lack of fine-scale resolution can be attributed to the temporal coarseness of EPA measurements, which are only available as 1-h integrated averages (Fig. 2G), and sparsity of regulatory monitoring sites, such that interpolation depended on measurements made by monitors located 4–8 km from where exposure took place (Fig. 2A).

## Discussion

Portable air pollution sensors can be used to measure personal exposures directly and at relatively low cost, making them an attractive tool to overcome some of the limitations posed by traditional methods of exposure assessment. In our semi-structured interviews with adults with persistent asthma, all participants stated they would be willing to use a sensor to measure their personal exposure to air pollution, and most expressed willingness to share sensor data with healthcare providers, researchers and the general public. In addition, our field trials deploying pollution sensors for real-time mobile monitoring of PM_2.5_ demonstrated their utility for capturing personalized air quality information at high spatiotemporal resolution. In contrast to EPA measurements, which were only available as 1-h integrated averages at discrete monitoring sites, mobile sensor measurements were taken every second, allowing them to resolve fine-scale fluctuations in PM_2.5_ concentrations over time and space.

While personal exposure to air pollution is moderately correlated with proxy measures obtained using outdoor monitors over large timescales (e.g., 24-h averages) [49, 50], indirect measures are unlikely to capture short-term fluctuations in personal exposures given individual activity and mobility patterns and the significant spatiotemporal variability of air pollution concentrations [51]. Even acute air pollution exposure has been shown to decrease lung function and increase airway inflammation among healthy adults and adults with asthma [52, 53], and emerging research linking personal sensor data to physiological and clinical outcomes suggest that periodic exposure to polluted microenvironments can impact health. For example, a recent study comparing schoolchildren’s particulate exposure in home, school and commute microenvironments determined that exposure experienced while commuting was most strongly associated with albuterol use and urinary leukotriene E4, a biomarker for airway inflammation [26].

Findings from our sensor field trials are consistent with other studies that have demonstrated the suitability of sensors for personal pollution monitoring [25–31], though our interviews with adults with asthma highlight some of the practical hurdles impeding their use in large-scale health exposure studies [15]. All interviewed participants stated they would be willing to use a portable pollution sensor, but the consensus was that this device should be unobtrusive and inconspicuous. Some preferred a device that resembled jewelry, while others wanted pollution sensing to be embedded directly into their existing smartphone or smartwatch. Contrary to these preferences, current commercially available sensors are palm-sized or larger and relatively conspicuous (Supp. Table 1; Supp. Figure 5). Due to the cumbersome nature of most pollution sensors, participants of personal exposure studies are required to carry sensors in custom-made backpacks [25–29] or vests [32]. Study participants who are allowed to carry sensors in their own bags must be instructed to keep sensor inlets exposed to air to ensure proper measurement [31].

The need to keep sensors charged and connected to mobile devices via Bluetooth imposes an additional burden on study participants. A recent pilot study using AirBeam2 devices to monitor particulate pollution exposure in women undergoing fertility treatment reported that most study subjects had trouble keeping devices charged and frequently lost connectivity during the 3-day study period [54]. Research staff operating AirBeam sensors in the present study reported similar issues with spotty Bluetooth connections, although battery life did not pose a problem due to shorter deployment times (1–3 h). Due to the high participant burden associated with personal monitoring, personal exposure studies have collected at most one week of continuous data per participant and have typically enrolled fewer than 100 participants, with the largest cohort to date comprising 167 pregnant women and 183 children [55].

Concerns related to data privacy and sharing, an important issue raised by reviews of personal sensing and other mobile health technologies [56, 57], were not found among our interview participants. Most (13 of 15) participants were willing to share sensor data, including location, with researchers and healthcare providers, while a subset (n = 8) was willing to share sensor data with the general public. Implicit in the preferences of those willing to share sensor data with key personnel but not the general public is trust in the data security maintained by the research team or health care system. Especially given recent findings that many mobile health applications do not follow standard privacy and security practices [58, 59], it is imperative that researchers maintain the trust of study participants by clearly communicating during the consent process what kind of data is collected, who will view the data and how it will be protected. Throughout the study, patient-collected data must be transferred securely and kept on protected servers, with special care paid to location data.

There are limitations to our study. Because we did not calibrate AirBeam sensors prior to each deployment, we are unable to quantify their accuracy relative to research-grade sensors or adjust measures to ensure they more closely match true PM_2.5_ values. While the goal of our study was not to quantify pollution measures with precision, it is important to bear in mind that researchers who seek to do so must calibrate sensors before deployment and at regular intervals throughout use to ensure accurate measurement [15, 60]. Consumer air quality monitors like the AirBeam sensor used are nevertheless useful to capture relative spikes in PM_2.5_ concentration [44]. Additionally, because sensors are sensitive to climactic and environmental conditions, including humidity, temperature and particulate composition, calibrating devices with respect to these variables – usually via collocation with a reference monitor – can considerably improve accuracy and reliability [43]. Careful consideration of these additional variables should be included in studies that link sensor measures to health outcomes.

Our interview participants were residents of relatively few zip codes in the Greater Philadelphia Area, and it is unclear whether attitudes and preferences regarding sensor use might vary across different neighborhoods. In addition, participants consisted of relatively few adults with asthma, the majority of whom were female (13/15) and non-Hispanic Black (11/15), consistent with the characteristics of people with asthma encountered most frequently by our health system. Because studies conducted in other patient groups and the general population have found that men and people who were White, had higher educational attainment or earned higher incomes were more willing to share mobile health and geolocation data for research purposes than others [61–63], we expect that our results on willingness to share sensor data would generalize to adults with asthma who are men and/or belong to other racial/ethnic groups. However, more work is needed to understand how privacy preferences regarding sensor data might differ among asthma patients across gender, racial/ethnic and socioeconomic strata.

## Conclusions

Patients with persistent asthma expressed interest in using sensors to monitor their personal exposure to PM_2.5_ and were generally willing to share personal sensor data with researchers and healthcare providers. Compared to traditional methods of personal exposure assessment, low-cost sensors captured personalized air quality information at higher spatiotemporal resolution. Improvements to currently available sensors, including more reliable Bluetooth connectivity, effortless portability, and longer battery life would facilitate their use in a general patient population.

## Supplementary Information

**Additional file 1: Supplemental Figure 1. **Photograph of a HabitatMap AirBeam sensor paired via Bluetooth to an Android mobile phone through the AirCasting app. **Supplemental Figure 2. **Results from an indoor test of basic sensor functionality and inter-sensor reliability. A) PM2.5 concentration measured by different AirBeam sensors, colored by sensor ID, while a stick of burning incense emitted particles in a confined indoor space. The dotted black line indicates the time at which the incense was lit. B) Correlation matrix of PM2.5 measurements recorded by different sensors. **Supplemental Figure 3. **Scatterplot of PM2.5 concentration measured by Airbeam sensors vs. PM2.5 concentration interpolated from space- and time-matched EPA measurements. **Supplemental Figure 4. **Time trends of temperature, relative humidity and PM2.5 concentration measured by an AirBeam sensor while being deployed outside on July 12, 2018. **Supplemental Figure 5. **Photographs of select commercially available particulate pollution sensors labeled by model name (manufacturer), with asterisks denoting those suitable for personal sensing. For sensor specifications, refer to **Supplemental Table 1**. **Appendix. **Standardized script used to guide semi-structured interviews with adults with asthma.

## Data Availability

The datasets used and analysed during the study are available from the corresponding author on reasonable request.
