# Supplementary material for: Feasibility and acceptability of monitoring personal air pollution exposure with sensors for asthma self-management
Source: Asthma Res Pract. 2021 Sep 5;7:13. doi: 10.1186/s40733-021-00079-9 (PMC8420032; doi:10.1186/s40733-021-00079-9)
Supplement: Supplementary file 1 — Additional file 1: Supplemental Figure 1. Photograph of a HabitatMap AirBeam sensor paired via Bluetooth to an Android mobile phone through the AirCasting app. Supplemental Figure 2. Results from an indoor test of basic sensor functionality and inter-sensor reliability. A) PM2.5 concentration measured by different AirBeam sensors, colored by sensor ID, while a stick of burning incense emitted particles in a confined indoor space. The dotted black line indicates the time at which the incense was lit. B) Correlation matrix of PM2.5 measurements recorded by different sensors. Supplemental Figure 3. Scatterplot of PM2.5 concentration measured by Airbeam sensors vs. PM2.5 concentration interpolated from space- and time-matched EPA measurements. Supplemental Figure 4. Time trends of temperature, relative humidity and PM2.5 concentration measured by an AirBeam sensor while being deployed outside on July 12, 2018. Supplemental Figure 5. Photographs of select commercially available particulate pollution sensors labeled by model name (manufacturer), with asterisks denoting those suitable for personal sensing. For sensor specifications, refer to Supplemental Table 1. Appendix. Standardized script used to guide semi-structured interviews with adults with asthma. [file 40733_2021_79_MOESM1_ESM.pdf]

Feasibility and Acceptability of Monitoring Personal Air Pollution Exposure with Sensors for Asthma Self-Management

SUPPLEMENTARY MATERIALS

Table of Contents

Supplemental Figure 1. Photograph of a HabitatMap AirBeam sensor paired via Bluetooth to an Android mobile phone through the AirCasting app.

Supplemental Figure 2. Results from an indoor test of basic sensor functionality and inter-sensor reliability. A) $PM_{2.5}$ concentration measured by different AirBeam sensors, colored by sensor ID, while a stick of burning incense emitted particles in a confined indoor space. The dotted black line indicates the time at which the incense was lit. B) Correlation matrix of $PM_{2.5}$ measurements recorded by different sensors.

Supplemental Figure 3. Scatterplot of $PM_{2.5}$ concentration measured by Airbeam sensors vs. $PM_{2.5}$ concentration interpolated from space- and time-matched EPA measurements.

Supplemental Figure 4. Time trends of temperature, relative humidity and $PM_{2.5}$ concentration measured by an AirBeam sensor while being deployed outside on July 12, 2018.

Supplemental Figure 5. Photographs of select commercially available particulate pollution sensors labeled by model name (manufacturer), with asterisks denoting those suitable for personal sensing. For sensor specifications, refer to Supp. Table 1.

Appendix: Standardized script used to guide semi-structured interviews with adults with asthma.

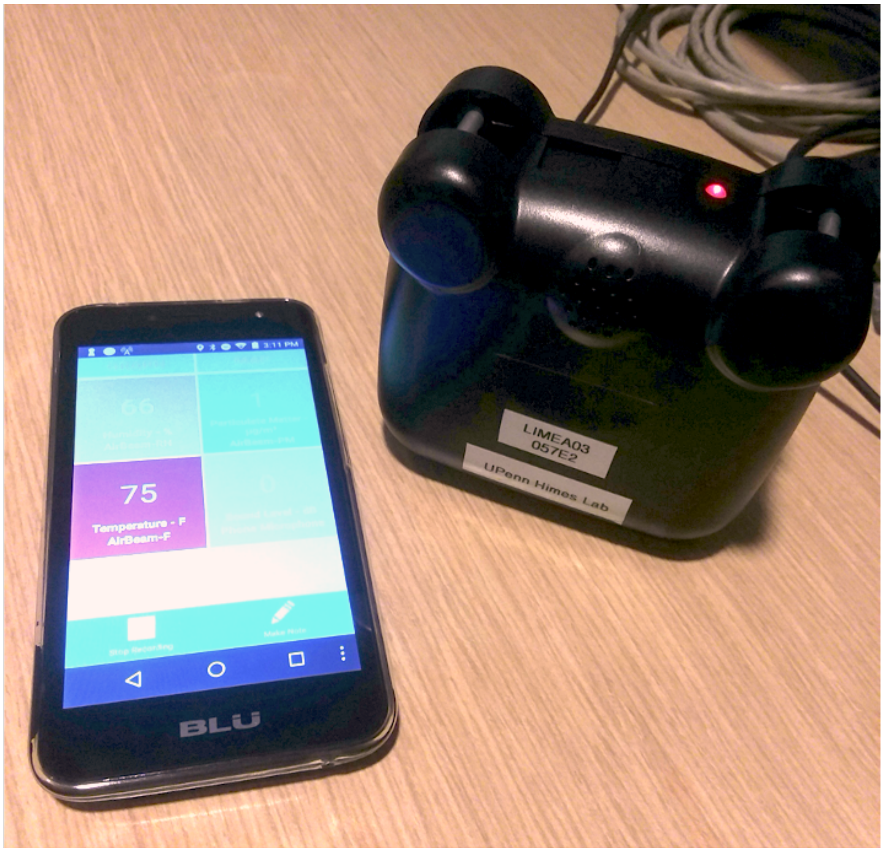

Supplemental Fig. 1. Photograph of a HabitatMap AirBeam sensor paired via Bluetooth to an Android mobile device through the AirCasting app

A

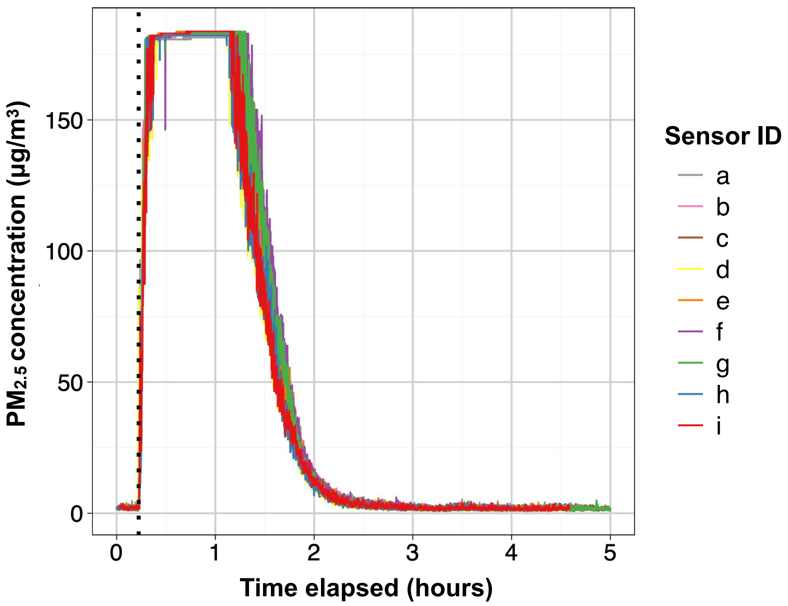

B

	a	b	c	d	e	f	g	h	i
a	1.000								
b	0.997	1.000							
c	0.997	0.998	1.000						
d	0.991	0.992	0.994	1.000					
e	0.998	0.997	0.997	0.991	1.000				
f	0.998	0.996	0.995	0.987	0.997	1.000			
g	0.998	0.997	0.997	0.989	0.998	0.998	1.000		
h	0.993	0.995	0.997	0.996	0.994	0.989	0.993	1.000	
i	0.994	0.995	0.997	0.996	0.995	0.990	0.993	0.997	1.000

Supplemental Fig. 2. Results from an indoor test of basic sensor functionality and inter-sensor reliability. A) $PM_{2.5}$ concentration measured by different AirBeam sensors, colored by sensor ID, while a stick of burning incense emitted particles in a confined indoor space. The dotted black line indicates the time at which the incense was lit. B) Correlation matrix (showing Pearson's *r*) of $PM_{2.5}$ measurements recorded by the different sensors.

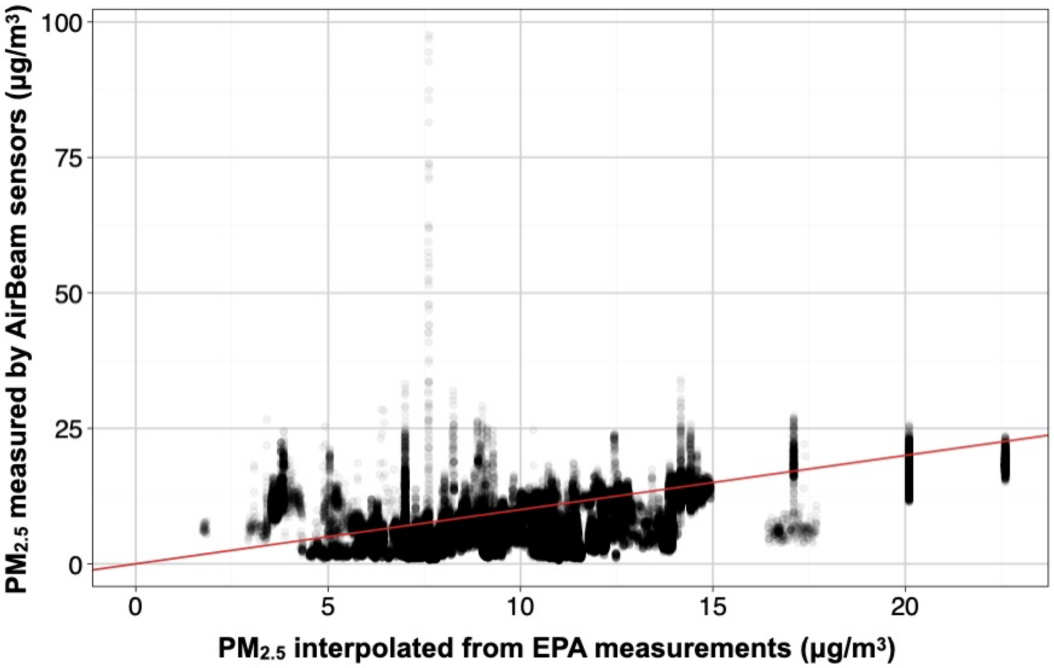

Supplemental Fig. 3. Scatterplot of $PM_{2.5}$ concentration measured by AirBeam sensors vs. $PM_{2.5}$ concentration interpolated from space- and time-matched EPA measurements

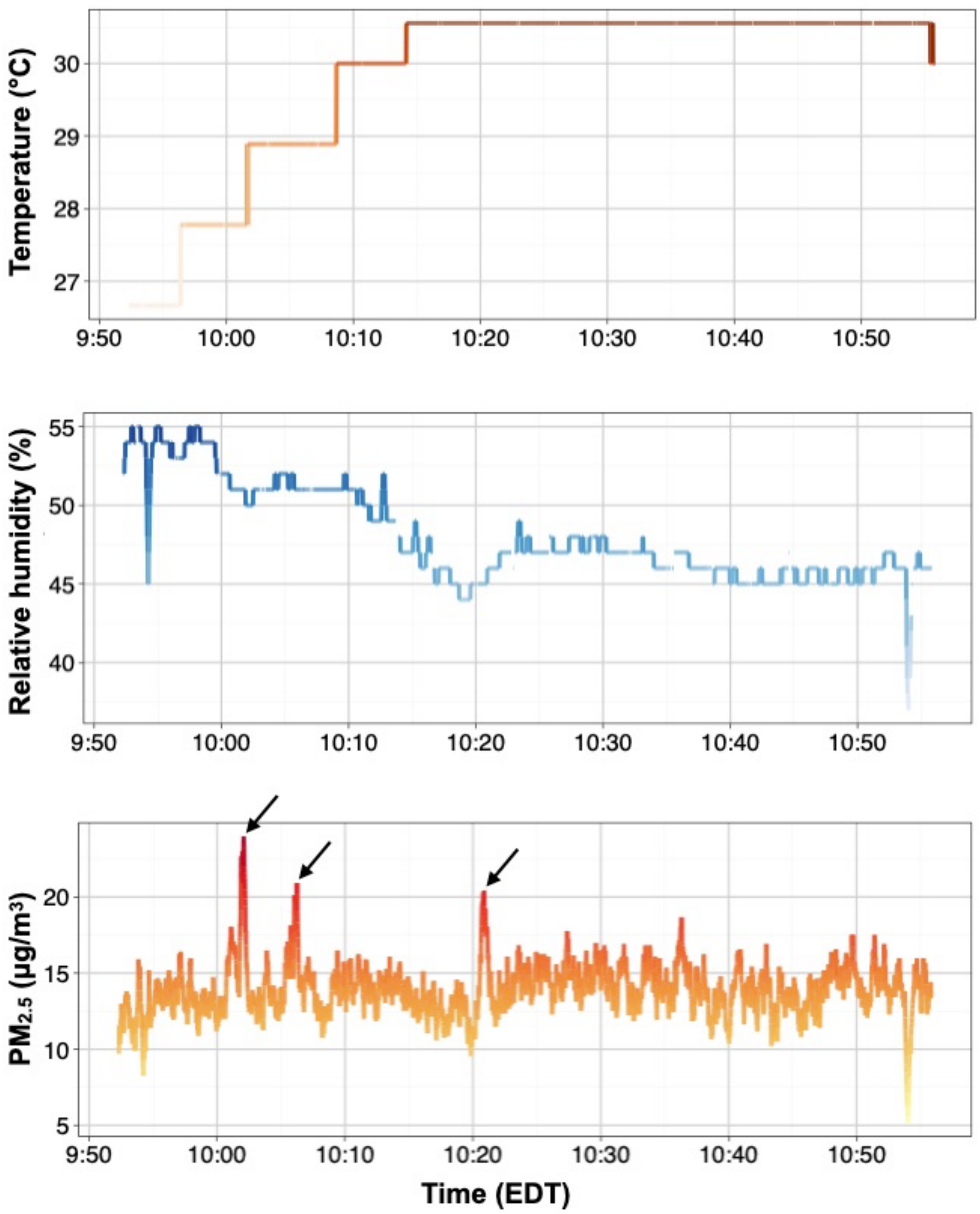

Supplemental Fig. 4. Time trends of temperature, relative humidity and $PM_{2.5}$ concentration measured by an AirBeam sensor while being deployed outside on July 12, 2018. Arrows indicate transient spikes in $PM_{2.5}$ concentration.

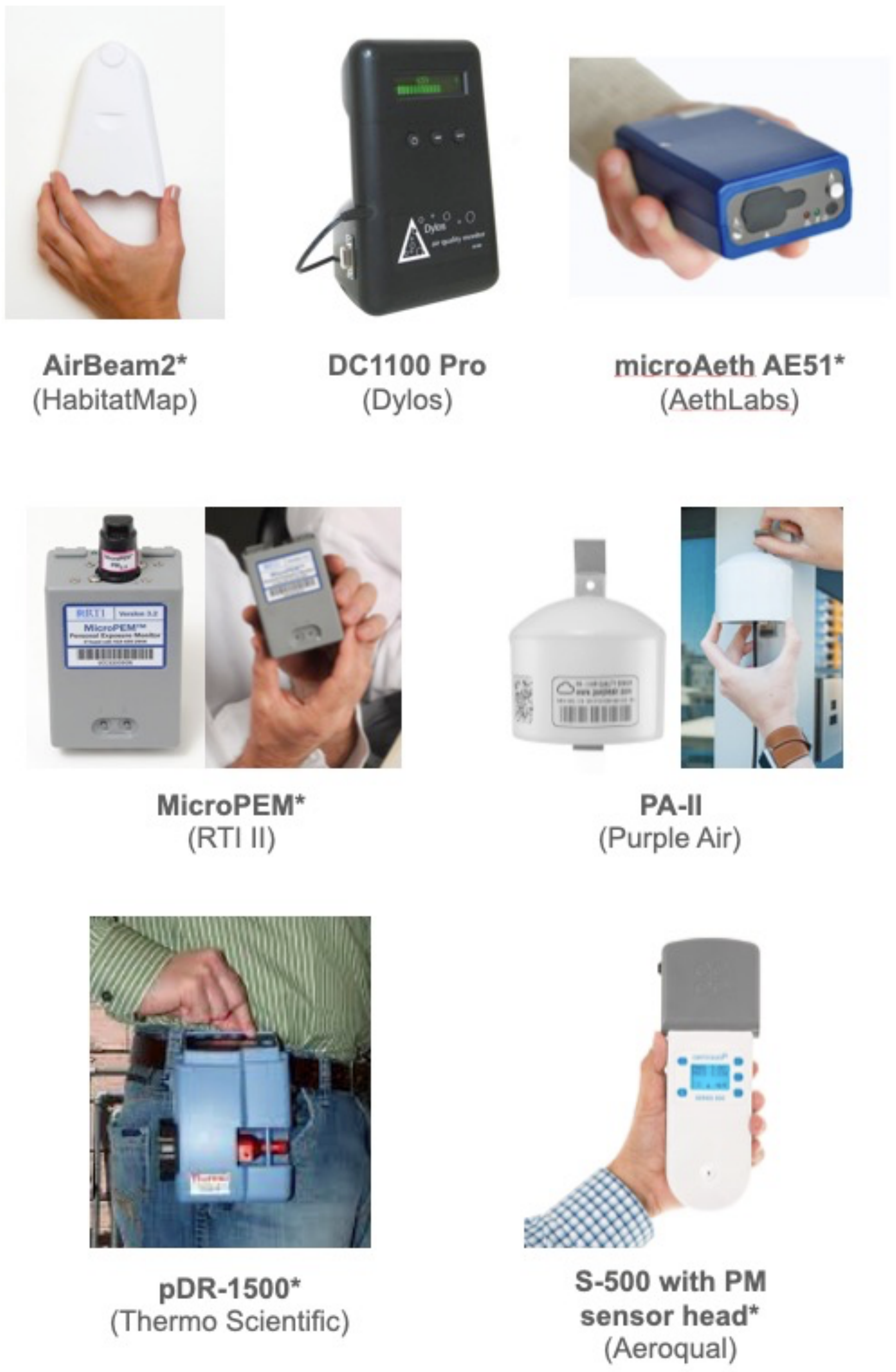

Supplemental Fig. 5. Photographs of select commercially available particulate pollution sensor labeled by model name (manufacturer), with asterisks denoting those suitable for personal sensing. For sensor specifications, refer to Supp. Table 1.

Appendix: Standardized script used to guide semi-structured interviews with adults with asthma.

Phone Interview Guide

Note: *This document serves as a guide for the interviewer. Interviewer discretion in phrasing, using probes and additional questions or explanation may be necessary because this is a qualitative study. Qualitative studies necessarily have a conversational aspect and will almost always diverge at some points from a script. The interviewer may therefore adjust wording, for instance, to acknowledge and take into account that the subject has already offered some information in response to a prior question, to clarify a response, or to solicit more information.*

INTRODUCTION

- Hello, thank you for agreeing to this interview today and for taking time out of your day to speak with me. ***My name is _________, and I am a _________ at the University of Pennsylvania.*** Our goal in interviewing you today is to understand whether portable pollution sensors can be used to monitor air quality by people with asthma, and if so, what information might be helpful to patients to receive as feedback.
- We will use your insights along with those of other people we interview, while maintaining your confidentiality, to help design future studies that use portable pollution devices and health information technologies to help patients manage their disease.
- We asked you to participate because you are a person with asthma who takes inhaled steroids, lives in the greater Philadelphia area (*Counties: Philadelphia, Delaware, Chester, Montgomery, Bucks, Camden, Gloucester, or Burlington*) and seeks care within the University of Pennsylvania Health System.

Before we start, I'd like to confirm that you are comfortable with recording this conversation. Is this correct? (Pause for confirmation). Recording the interview is to make sure that I don't miss anything you say. I may also take a few notes while we talk. Please know that everything you say today is confidential. The things you share will not be connected to your name, and the recording will be destroyed after it is transcribed. Any identifying information, like your name or your doctors' names, will be removed from that transcript. If you need a break or want to stop at any point, please let me know. Do you have any questions before we start? (Pause for questions.)

INTERVIEW

Turn on Tape Recorder
I am turning the recorder on now.

Interviewer should state date, interviewer initials, participant initials or ID into recorder.

Interview begins, make sure people are given time to think before answering the questions and don't move too quickly. Use the probes to make sure that all issues are addressed, but move on when you feel you are starting to hear repetitive information.

Questions:

- Icebreaker: How long have you lived in your neighborhood?

- Icebreaker: How have things changed since you have lived here? (*Prompts: Buildings/construction, people, traffic, stores, etc.)*

- Icebreaker: How long have you had asthma?

    - Can you tell me about when you were first diagnosed?
    - How has it affected you since then?
    - Have there been times when your asthma is better? Worse?

- Tell me about when your asthma symptoms get worse. (*Rephrase option:* What are some of the triggers, or things that make your asthma symptoms get worse?)

    - How did you identify your asthma triggers?
    - What, if anything, do you do to avoid your triggers?
    - Does avoiding your triggers help you manage your asthma?
    - If you could give someone advice on how to avoid their asthma triggers, what would it be?
    - In an ideal world, how would your asthma triggers be avoided?
    - How do you talk with your provider about triggers for your asthma?
    - How do you talk with your provider about the environmental triggers (*such as smoke, dust, gas/diesel fumes, smog, etc.)* for your asthma?
    - What information or guidance, if any, have they shared?
    - Anything you would like them to share?

- What do you think about air quality?

    - What would you like to know about it?
    - Do you feel the air quality in your neighborhood has changed? The air quality around you as you go about your day-to-day life?
    - In an ideal world, what could be done to improve air quality?

- What do you think about air pollution around you?

    - Does it affect your asthma symptoms?
    - Do you do anything to avoid it?
    - Do you keep track of pollution or air quality?
    - Do you use any technology, such as an app or a website, to track or measure your asthma or general health?
        - If yes, what do you use? What do you like about it?

    - If no, would you be interested in using one? What information might be useful for you to track?

Introduce the device: There are small devices, that range in size from a watch to a book, that use sensors to measure things like air quality and pollution.

- What do you think of using such a device to measure air quality in your home? At work?

    - What would you want this device to look like to make it something you would consider using?
    - If you could decide what information the device provided, what things would be good to include?
        - Would you want it to provide pictures or text?
        - How often would you want to receive that information?
    - How would this device be helpful to you in managing your asthma?

- What would you think if you had a device that you could carry with you, say by attaching to your clothes or a bag/backpack, that provided information about air quality and pollution near you?

    - Would you consider wearing it to find out how much pollution there is wherever you go?
    - If you had to charge the device every day, like a cell phone, would that impact your decision?
    - What, if anything, would make you not want to use such a device?

- If you were to gather information about pollution near you using a portable device, who would you be comfortable sharing this information with? (*Probe:* Health providers, other patients, the general public?)

    - Is there anyone who you would not be comfortable sharing this information with?
    - If this information could be helpful to your neighbors or researchers, say by alerting them to when pollution is high or by helping to identify sources of pollution, how would that impact who you'd be comfortable sharing the information with?

    - Would you consider sharing information you have collected via Social Media (such as Twitter, Instagram, Facebook, Snapchat, etc)? (If subjects are not familiar with social media or the patient portal, ask to what extent they'd be interested in learning to use them (i.e., Twitter, Facebook, Instagram, Patient Portal).
    - Would you consider sharing it via a patient portal that only your health providers could see?
    - How do you think using these platforms would be helpful to others?

    - If this information existed already (*i.e., not specifically collected by the participant),* is there anyone you feel should or should not have access to this type of information?

Topics to probe about:

  - If you were to use a portable device, some of these track location with GPS, although they can't tell who a person is with or what they are doing. How do you feel about a sensor tracking your location?
    - If the information does not contain your name or personal information?
    - If the information was summarized to the level of a city block or neighborhood?
    - If the information was summarized over time – hours, days, week?

- What would you think about sharing pictures of your home to a health provider or research group so that they can better understand your environment?

  - What about pictures of your neighborhood?
  - In what ways might you be comfortable sharing this information with your provider or a research group (i.e. patient portal, print out and bring in, email, etc.)?

- Is there anything I didn't ask you about that you want to share on the topic of pollution or other triggers affecting your asthma?

CONCLUSION

That concludes our interview. Thank you so much for sharing your thoughts and opinions with me. I'm going to turn off the tape recorder now.

Turn off Tape Recorder

Notify subject that they will receive a $30 incentive to thank them for their participation.
